# Higher titer hepatitis B core antibody predicts a higher risk of liver metastases and worse survival in patients with colorectal cancer

**DOI:** 10.1186/s12957-021-02369-1

**Published:** 2021-08-26

**Authors:** Ziyao Li, Shaofei Li, Hangbo Tao, Yixiang Zhan, Kemin Ni, Jianfeng Gong, Guoxun Li

**Affiliations:** 1 Department of Colorectal Surgery, Tianjin Union Medical Center, No. 190 Jieyuan street, Hongqiao District, Tianjin, 300121 China; 2 School of Medicine, Nankai University, Tianjin, 300071 China; 3 Department of General Surgery, Jinling Hospital, Medical School of Nanjing University, Nanjing, China

**Keywords:** Hepatitis B virus, Hepatitis B core antibody, Colorectal cancer, Colorectal liver metastases

## Abstract

**Background:**

There have been controversial voices on if hepatitis B virus infection decreases the risk of colorectal liver metastases or not. This study aims to the find the association between HBV infection and postoperative survival of colorectal cancer and the risk of liver metastases in colorectal cancer patients.

**Methods:**

Patients who underwent curative surgical resection for colorectal cancer between January 2011 and December 2012 were included. Patients were grouped according to anti-HBc. Differences in overall survival, time to progress, and hepatic metastasis-free survival between groups and significant predictors were analyzed.

**Results:**

Three hundred twenty-seven colorectal cancer patients were comprised of 202 anti-HBc negative cases and 125 anti-HBc positive cases, and anti-HBc positive cases were further divided into high-titer anti-HBc group (39) and low-titer anti-HBc group (86). The high-titer anti-HBc group had significantly worse overall survival (5-Yr, 65.45% vs. 80.06%; *P* < .001), time to progress (5-Yr, 44.26% vs. 84.73%; *P* < .001), and hepatic metastasis-free survival (5-Yr, 82.44% vs. 94.58%; *P* = .029) than the low-titer group. Multivariate model showed anti-HBc ≥ 8.8 S/CO was correlated with poor overall survival (HR, 3.510; 95% CI, 1.718–7.17; *P* < .001), time to progress (HR, 5.747; 95% CI, 2.789–11.842; *P* < .001), and hepatic metastasis-free survival (HR, 3.754; 95% CI, 1.054–13.369; *P* = .041) in the anti-HBc positive cases.

**Conclusions:**

Higher titer anti-HBc predicts a potential higher risk of liver metastases and a worse survival in anti-HBc positive colorectal cancer patients.

**Supplementary Information:**

The online version contains supplementary material available at 10.1186/s12957-021-02369-1.

## Background

Colorectal cancer (CRC) is the third most common carcinoma in the world [1]. The 5-year relative survival rate for CRC is 65% [2]. Distant metastases are major cause of death and liver is the most common metastatic site [3].

Inflammation has close relationship with the forming of tumor. In the nineteenth century, German doctor Rudolf Virchow [4] first observed leukocytes infiltration in tumor tissue. Then, it was expounded that the leukocytes infiltrated in tumor tissue could generate inflammatory mediators, thereby forming inflammatory microenvironment in tumor tissue which was further founded to stimulate the development of tumor [5–7]. Chronic inflammation could be sustained by the long-term activity of harmful microorganisms in the human body [8, 9], which may promote tumor formation and development.

HBV is the virus that leads to inflammation and necrosis of hepatocytes during infection, and it is one of the leading public health problems in China [10]. As a consequence, there are a large number of CRC patients accompanied by HBV. However, whether HBV infection affects the incidence of colorectal liver metastases (CRLM) remains unclear.

As almost all of the patients generate hepatitis B core antibody (anti-HBc) after HBV infection, hepatitis B core antibody (anti-HBc) is recognized as the most sensitive serum marker [11] in the course of HBV infection. Therefore, anti-HBc was used as a marker of recent or previously infection of HBV. Furthermore, as the titer of anti-HBc showed positive correlation with the activity of HBV replication [12, 13], the relationship between HBV replication and outcomes of the crowd in the study was also analyzed.

## Methods

### Patients

A retrospective analysis was conducted by reviewing the clinicopathological data of CRC patients who underwent curative resection from January 2011 to December 2012 at the Tianjin Union Medical Center. Patients who met the following criteria were enrolled: (1) histologically confirmed colorectal malignancy, (2) TNM stage II or III, (3) no evidence of distant metastases was found before the operation, (4) R0 resection for primary lesion, and (5) aged between 40 and 75 years. All clinicopathological data, including clinicopathological features, tumor characteristics, and laboratory examinations, were obtained from medical records and follow-up system of Tianjin Union Medical Center. All the patients included were staged according to AJCC Cancer Staging Manual, 7th edition.

### Treatment

Patients with colon cancer received imaging examinations consisting of thoracic, abdominal, and pelvic computed tomography (CT), abdominal ultrasound, abdominal and/or pelvic magnetic resonance imaging (MRI) (if necessary), and colonoscopy before operation.

Patients with rectal cancer received imaging examinations consisting of thoracic, abdominal, and pelvic CT, abdominal ultrasound, pelvic MRI, abdominal MRI (if necessary), and colonoscopy before operation.

The primary colorectal malignancy was radically resected from all eligible patients according to the principle of total mesorectal excision (TME) or complete mesocolic excision (CME). Depending on patients’ conditions, neoadjuvant therapy and adjuvant treatment was determined by the multidisciplinary team (MDT). For colon cancer, neoadjuvant FOLFOX or CAPEOX was considered for bulky nodal disease or clinical T4b; neoadjuvant radiation therapy (RT) with concurrent fluoropyrimidine (FU)-based chemotherapy was considered for initially unresectable or medically inoperable tumor to aid resectability; adjuvant treatment consisting FOLFOX, or CAPEOX, or capecitabine alone, or 5-FU/leucovorin, or observation was considered according to the pathologic stage. For the rectal cancer, neoadjuvant therapy consisting long-course chemotherapy/RT or short-course RT was considered according to clinical stage; FOLFOX, or CAPEOX, or infusional 5-FU/RT, or capecitabine/RT followed by FOLFOX or CAPEOX, or observation was considered for adjuvant treatment according to pathologic stage and clinical stage.

### Group

All of the cases were grouped based on anti-HBc. Firstly, all cases were grouped into anti-HBc positive group and anti-HBc negative group. Moreover, the anti-HBc positive group was grouped into the high-titer anti-HBc group and the low-titer anti-HBc group by the cutoff point of 8.8 S/CO which was calculated based on time to progress (TTP).

### Serologic assay for CRC patients

All laboratory results, including biochemical tests, serum tumor markers, and HBV infection tests, were obtained within 1 week before the operation. Hepatitis B surface antigen (HBsAg), antibodies to hepatitis B surface antigen (anti-HBs), and anti-HBc were detected by electrochemical luminescence. Carcinoembryonic antigen (CEA) levels above 5 ng/ml, carbohydrate antigen 19-9 (CA19-9) levels above 37 U/ml, and anti-HBc levels above 1S/CO were considered to be elevated.

### Follow-up of patients

All patients were followed up after hospital discharge. The patients were followed up every 3 months in the first 2 years after surgery and then semi-annually during the third to the fifth year. The follow-up evaluation included a routine physical, blood test, tests for the tumor markers CEA and CA19-9, and abdominal ultrasonography. Thoracic, abdominal, and pelvic computed tomography (CT) were performed annually. Colonoscopy was performed in 1 year after surgery except if no preoperative colonoscopy due to obstructing lesion, colonoscopy in 6 months. If advanced adenoma was founded, repeat it in 1 year; otherwise, repeat it in 3 years, then every 5 years. Magnetic resonance imaging (MRI) was performed when necessary. The follow-up period was terminated in July 2019.

### Non-invasive prediction methods calculation formulae

The following fibrosis 4 score (FIB-4) equations was used to evaluate the extent of fibrosis [14, 15]. Calculation formulae of FIB-4:$\mathrm{FIB}-4=\frac{\mathrm{Age}\left(\mathrm{years}\right)\times \mathrm{AST}\left(\mathrm{U}/\mathrm{L}\right)}{\mathrm{Platelets}\left({10}^9/\mathrm{L}\right)\times \sqrt{\mathrm{ALT}\left(\mathrm{U}/\mathrm{L}\right)}}$ [15]; neutrophil to lymphocyte ratio (NLR) was used to indicate the extent of inflammatory response.$\mathrm{NLR}=\frac{\mathrm{Neutrophil}\left({10}^9/\mathrm{L}\right)}{\mathrm{Lymphocyte}\left({10}^9/\mathrm{L}\right)}$ [16]. The cutoff points for FIB-4 and NLR are 1.45 and 3.4, respectively.

### Statistical methods

Continuous variables are presented as means ± standard deviation (SD). Categorical variables are shown as the number of cases and percentages. Comparisons for continuous variables were performed using Student’s *t* test or Mann-Whitney *U* test. Chi-square test and Fisher’s exact test was performed for the categorical variables. Overall survival (OS), TTP, and hepatic metastasis-free survival (HMFS) outcomes were compared using Kaplan-Meier curves. Log-rank test was used to determine statistical differences between curves. Univariate and multivariate analyses were performed by Cox proportional hazards regression models to determine the hazard ratio of each factor. Variables that were showed a significant univariate relationship with outcome were entered into the multivariate analysis. OS was defined from the date of surgery to the date of death or last follow-up. TTP was defined from the date of surgery to the date of disease progression. HMFS was defined from the date of surgery to the date of occurrence of hepatic metastases. The optimal cutoff point of NLR and anti-HBc are determined by X-tile 3.6.1 software (Yale University, New Haven, CT, USA) based on TTP. All statistical analyses were performed using SPSS 22.0 statistical software (IBM, NY, USA) and GraphPad Prism version 8.01 (GraphPad Software, Inc., La Jolla, CA, USA). A two-tailed *P* value < .05 was interpreted as statistically significant.

## Results

### Baseline characteristics of patients

A total of 327 cases were qualified for the analyses. Among them, 202 (61.8%) cases were anti-HBc negative and 125 (38.2%) cases were anti-HBc positive including 8 (2.4%) HBsAg positive cases. The 125 anti-HBc positive cases were divided into two groups according to optimal cut-off point of anti-HBc titer (8.8 S/CO): 39 (31.2%) cases were classified into the high-titer anti-HBc group, while the remaining 86 (68.8%) cases were classified into the low-titer anti-HBc group. The comparisons of baseline characteristics were shown in Table 1. No statistical difference between the anti-HBc positive and negative group was identified except for the gender proportion in which the proportion of male was significantly higher in the anti-HBc positive group than the anti-HBc negative group (68% vs. 55.4%, *P* = .027). Besides, no significant difference was identified between the high-titer and low-titer anti-HBc group.

**Table 1:** Characteristics of the included patients

Variable	anti-HBc positive, *n* (%)	anti-HBc negative, *n* (%)	*P* value	High-titer anti-HBc, *n* (%)	Low-titer anti-HBc, *n* (%)	*P* value
*N*	125	202		39	86	
Age (years)	61.32 ± 8.01	60.61 ± 7.55	0.419	59.44 ± 7.469	62.17 ± 8.147	0.077
Gender			0.027*			0.154
Male	85 (68)	112 (55.4)		23 (59.0)	62 (72.1)	
Female	40 (32)	90 (44.6)		16 (41.0)	24 (27.9)	
Tumor site			0.72			1
Colon	44 (35.2)	67 (33.2)		14 (35.9)	30 (34.9)	
Rectum	81 (64.8)	135 (66.8)		25 (64.1)	56 (65.1)	
T stage			0.458			0.523
T1	1 (0.8)	1 (0.5)		0 (0)	1 (1.2)	
T2	4 (3.2)	9 (4.5)		0 (0)	4 (4.7)	
T3	120 (96)	188 (93.1)		39 (100)	81 (94.2)	
T4	0 (0)	4 (2)		0 (0)	0 (0)	
N stage			0.288			0.202
N0	65 (52)	121 (59.9)		20 (51.3)	45 (52.3)	
N1	41 (32.8)	60 (29.7)		10 (25.6)	31 (36)	
N2	19 (15.2)	21 (10.4)		9 (23.1)	10 (11.6)	
TNM stage			0.167			1
II	65 (52)	122 (60.4)		20 (51.3)	45 (52.3)	
III	60 (48)	80 (39.6)		19 (48.7)	41 (47.7)	
Histological types			0.447			0.092
Adenocarcinoma	107 (85.6)	166 (82.2)		31 (79.5)	78 (90.7)	
Mucinous adenocarcinoma	18 (14.4)	36 (17.8)		8 (20.5)	8 (9.3)	
Differentiation			0.652			0.253
Well	6 (4.8)	10 (5)		1 (2.6)	5 (5.8)	
Moderately	87 (69.6)	152 (75.2)		29 (74.4)	58 (67.4)	
Poorly	19 (15.2)	22 (10.9)		3 (7.7)	16 (18.6)	
Not Available	13 (10.4)	18 (8.9)		6 (15.4)	7 (8.1)	
Neoadjuvant therapy			1			0.501
Yes	10 (8)	15 (7.4)		4 (10.3)	6 (7)	
No	115 (92)	187 (92.6)		35 (89.7)	80 (93)	
Retrieved LN			0.173			0.663
< 12	32 (25.6)	67 (33.2)		11 (28.2)	21 (24.4)	
≥ 12	93 (74.4)	135 (66.8)		28 (71.8)	65 (75.6)	
CEA (ng/ml)			0.909			0.332
≤ 5	70 (56)	111 (55)		19 (48.7)	51 (59.3)	
> 5	55 (44)	91 (45)		20 (51.3)	35 (40.7)	
CA19-9(U/ml)			0.492			0.27
≤ 37	107 (85.6)	179 (88.6)		31 (79.5)	76 (88.4)	
> 37	18 (14.4)	23 (11.4)		8 (20.5)	10 (11.6)	
ALT(U/L)	17.65 ± 9.50	19.86 ± 15.86	0.377	18.41 ± 9.85	17.31 ± 9.37	0.434
AST(U/L)	18.42 ± 6.70	19.01 ± 9.60	0.892	18.10 ± 6.12	18.56 ± 6.98	0.94
ALP(U/L)	83.90 ± 21.67	84.66 ± 24.98	0.86	82.56 ± 20.30	84.51 ± 22.34	0.643
PLT (1 × 10^9^/L)	252.90 ± 67.98	250.91 ± 77.43	0.622	261.97 ± 67.56	84.51 ± 22.34	0.317

*Abbreviations*: anti-HBc, antibodies to hepatitis B core antigen, *CA19-9* cancer antigen 19-9, *CEA* carcinoembryonic antigen, *ALT* alanine transaminase, *AST* aspartate transaminase, *ALP* alkaline phosphatase; PLT, platelets; LN, lymph node

*Significant at *P* < 0.05

### Overall survival, time to progress, and hepatic metastasis-free survival difference according to anti-HBc status

**Fig. 1:**
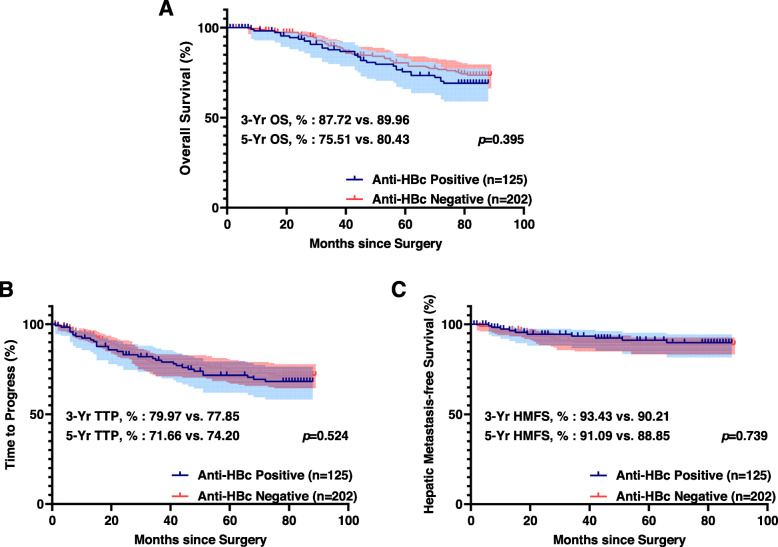
Kaplan–Meier curves of overall survival (OS), time to progress (TTP), and hepatic metastasis-free survival (HMFS) between the anti-HBc positive group and anti-HBc negative group. **A** Overall survival curve. **B** Time to progress curve. **C** Hepatic metastasis-free survival curve. The light blue and red area represent 95% confidence intervals of each group. There is no significant difference between the two groups

**Fig. 2:**
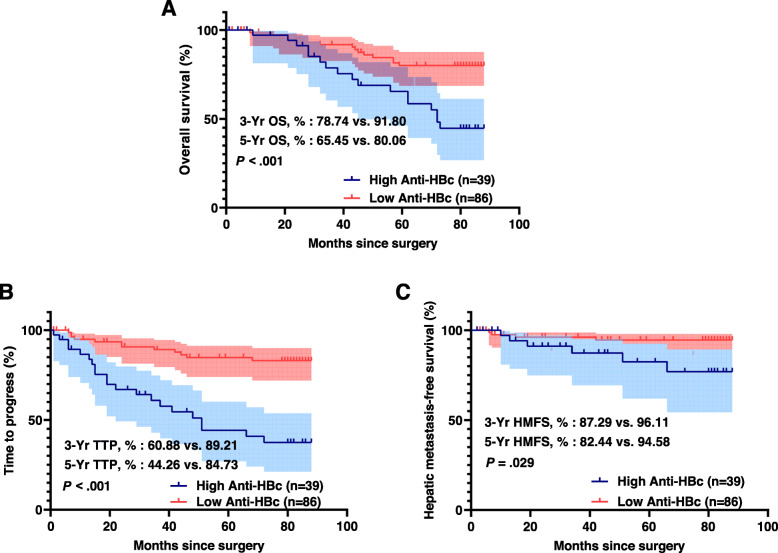
Kaplan–Meier curves of overall survival (OS), time to progress (TTP), and hepatic metastasis-free survival (HMFS) between the high-titer anti-HBc group and low-titer anti-HBc group. The light blue and red area represent 95% confidence intervals of each group. Patients in the high-titer anti-HBc group had shorter OS, TTP, and HMFS than those in the low-titer anti-HBc group

The mean follow-up period was 61.2 ± 28.8 months. Recurrence was observed in 84 (25.7%) of 327 patients until the last follow-up. There were 30 (9.2%) hepatic recurrences, 37 (11.3%) lung recurrences, 11 (3.36%) bone recurrences, 11 (3.36%) pelvic recurrences, and 5 (1.5%) instances of brain recurrences. The OS, TTP, and HMFS curves for the anti-HBc positive and negative groups are shown in Fig. 1. The 3-, 5- year OS (3-Yr, 87.72% vs. 89.96%; 5-Yr, 75.51% vs. 80.43%; *P* = .395; Fig. 1A), TTP (3-Yr, 79.97% vs. 77.85%; 5-Yr, 71.66% vs. 74.20%; *P* = .524; Fig. 1B), and HMFS (3-Yr, 93.43% vs. 90.21%; 5-Yr, 91.09% vs. 88.85%; *P* = .739; Fig. 1C) did not differ between the two groups. In contrast, there are significant differences identified between the high-titer and low-titer anti-HBc groups (Fig. 2). Patients in the high-titer anti-HBc group had worse OS (3-Yr, 78.74% vs. 91.80%; 5-Yr, 65.45% vs. 80.06%; *P* < 0.001; Fig. 2A), TTP (3-Yr, 60.88% vs. 89.21%; 5-Yr, 44.26% vs. 84.73%; *P* < 0.001; Fig. 2B), and HMFS (3-Yr, 87.29% vs. 96.11%; 5-Yr, 82.44% vs. 94.58%; *P* = .029; Fig. 2C) than those in the low-titer anti-HBc group.

### Prognostic factors for OS, TTP, and HMFS

Univariate and multivariate analyses of the prognostic factors for OS, TTP, and HMFS are presented in the Tables 2 and 3. For the CRC patients undergoing curative surgical resection, NLR ≥ 3.4 (HR, 1.838; 95% CI, 1.119–3.02; *P* = .016), CA19-9 > 37 U/ml (HR, 2.111; 95% CI, 1.229–3.624; *P* = .007), and stage III (HR, 3.511; 95% CI, 2.162–5.702; *P* < 0.001) were associated with worse OS; NLR ≥ 3.4 (HR, 1.783; 95% CI, 1.094–2.906; *P* = .02) and stage III (HR, 3.579; 95% CI, 2.247–5.699; *P* < .001) were associated with worse TTP; NLR ≥ 3.4 (HR, 2.231; 95% CI, 1.043–4.773; *P* = .039) and stage III (HR, 2.985; 95% CI, 1.394–6.391; *P* = .005) were associated with worse HMFS (Table 2). However, univariate and multivariate analyses for the anti-HBc positive CRC patients undergoing curative surgical resection revealed that anti-HBc ≥ 8.8 (HR, 3.510; 95% CI, 1.718–7.17; *P* = .001) and stage III (HR, 3.038; 95% CI, 1.423–6.484; *P* = .004) were associated with worse OS; anti-HBc ≥ 8.8 (HR, 5.747; 95% CI, 2.789–11.842; *P* < 0.001) and stage III (HR, 3.722; 95% CI, 1.752–7.908; *P* < .001) were associated with worse TTP; only anti-HBc ≥ 8.8 (HR, 3.754; 95% CI, 1.054–13.369; *P* = .041) was associated with worse HMFS (Table 3).

**Table 2:** Risk factors for overall survival, time to progress, and hepatic metastasis-free survival in the CRC patients undergoing curative surgical resection

Variable	Overall survival						Time to progress						Hepatic metastasis-free survival					
	Univariate analysis			Multivariate analysis			Univariate analysis			Multivariate analysis			Univariate analysis			Multivariate analysis		
	HR	95% CI	*P* value	HR	95% CI	*P* value	HR	95% CI	*P* value	HR	95% CI	*P* value	HR	95% CI	*P* value	HR	95% CI	*P* value
Anti-HBc																		
Positive	1.219	0.771–1.926	0.397				1.175	0.757–1.823	0.473				0.879	0.412–1.878	0.74			
Negative	Reference						Reference						Reference					
FIB-4																		
≥ 1.45	1.523	0.944–2.459	0.085				1.032	0.629–1.693	0.9				1.108	0.493–2.489	0.804			
< 1.45	Reference						Reference						Reference					
NLR																		
≥ 3.4	1.982	1.207–3.254	0.007*	1.838	1.119–3.02	0.016*	1.866	1.146–3.04	0.012*	1.783	1.094–2.906	0.02*	2.367	1.108–5.059	0.026*	2.231	1.043–4.773	0.039*
< 3.4	Reference						Reference						Reference					
Anti-HBs																		
Positive	1.122	0.695–1.81	0.638				1.037	0.652–1.648	0.879				0.965	0.442–2.107	0.929			
Negative	Reference						Reference						Reference					
HBsAg																		
Positive	2.48	0.905–6.793	0.077				2.195	0.803–5.999	0.125				2.927	0.697–12.295	0.143			
Negative	Reference						Reference						Reference					
Retrieved LN																		
≥ 12	0.715	0.451–1.132	0.153				0.789	0.506–1.232	0.297				1.565	0.671–3.647	0.3			
< 12	Reference						Reference						Reference					
Neoadjuvant therapy																		
Yes	0.808	0.326–2	0.644				1.213	0.585–2.514	0.604				1.285	0.39–4.236	0.681			
No	Reference						Reference						Reference					
CEA (ng/ml)																		
> 5	1.272	0.811–1.995	0.294				1.151	0.748–1.773	0.523				1.769	0.859–3.642	0.122			
≤ 5	Reference						Reference						Reference					
CA19-9(U/ml)																		
> 37	2.352	1.371–4.035	0.002*	2.111	1.229–3.624	0.007*	1.548	0.871–2.751	0.136				1.157	0.404–3.315	0.787			
≤ 37	Reference						Reference						Reference					
TNM stage																		
III	3.609	2.223–5.859	< 0.001*	3.511	2.162–5.702	< 0.001*	3.628	2.279–5.775	< 0.001*	3.579	2.247–5.699	< 0.001*	3.084	1.442–6.597	0.004*	2.985	1.394–6.391	0.005*
II	Reference						Reference						Reference					
Gender																		
Male	1.035	0.651–1.644	0.885				1.16	0.741–1.815	0.517				1.771	0.789–3.979	0.166			
Female	Reference						Reference						Reference					
Age (years)																		
≥ 61	0.996	0.635–1.561	0.985				0.899	0.584–1.383	0.628				0.652	0.314–1.354	0.252			
< 61	Reference						Reference						Reference					

*Abbreviations*: *anti-HBc* antibodies to hepatitis B core antigen, *anti-HBs* antibodies to hepatitis B surface antigen, *HBsAg* hepatitis B surface antigen, *NLR* neutrophil to lymphocyte ratio; CA19-9, cancer antigen 19-9; CEA, carcinoembryonic antigen; ALT, alanine transaminase; AST, aspartate transaminase; ALP, alkaline phosphatase; PLT, platelets

*Significant at *P* < 0.05

**Table 3:** Risk factors for overall survival, time to progress, and hepatic metastasis-free survival in the anti-HBc positive CRC patients undergoing curative surgical resection

Variable	Overall survival						Time to progress						Hepatic metastasis-free survival					
	Univariate analysis			Multivariate analysis			Univariate analysis			Multivariate analysis			Univariate analysis			Multivariate analysis		
	HR	95% CI	*P* value	HR	95% CI	*P* value	HR	95% CI	*P* value	HR	95% CI	*P* value	HR	95% CI	*P* value	HR	95% CI	*P* value
Anti-HBc																		
≥ 8.8	3.18	1.564–6.463	< 0.001*	3.51	1.718–7.17	< 0.001*	4.889	2.397–9.97	< 0.001*	5.747	2.789–11.842	< 0.001*	3.754	1.054–13.369	0.04*	3.754	1.054–13.369	0.04*
< 8.8	Reference																	
FIB-4																		
≥ 1.45	1.48	0.718–3.05	0.288				1.018	0.484–2.139	0.963				0.983	0.254–3.806	0.981			
< 1.45	Reference																	
NLR																		
≥ 3.4	1.418	0.634–3.171	0.395				1.41	0.635–3.129	0.398				1.753	0.453–6.783	0.416			
< 3.4	Reference																	
Anti-HBs																		
Positive	1.093	0.515–2.321	0.817				0.873	0.429–1.774	0.706				0.756	0.213–2.68	0.665			
Negative	Reference																	
HBsAg																		
Positive	2.296	0.802–6.572	0.121				2.154	0.755–6.141	0.151				3.877	0.821–18.311	0.087			
Negative	Reference																	
Retrieved LN																		
≥ 12	0.983	0.453–2.135	0.966				0.94	0.447–1.975	0.869				1.66	0.352–7.82	0.521			
< 12	Reference																	
Neoadjuvant therapy																		
Yes	0.735	0.175–3.083	0.674				1.544	0.542–4.395	0.416				0.043	0–729.474	0.527			
No	Reference																	
CEA (ng/ml)																		
> 5	1.286	0.636–2.602	0.484				0.911	0.453–1.831	0.793				3.352	0.867–12.965	0.08			
≤ 5	Reference																	
CA19-9(U/ml)																		
> 37	1.683	0.725–3.907	0.226				1.282	0.529–3.106	0.582				0.634	0.08–5.008	0.666			
≤ 37	Reference																	
TNM stage																		
III	2.731	1.285–5.802	0.01*	3.038	1.423–6.484	<0.001*	3.038	1.444–6.39	<0.001*	3.722	1.752–7.908	<0.001*	1.921	0.541–6.822	0.312			
II	Reference																	
Gender																		
Male	1.211	0.542–2.709	0.64				0.853	0.414–1.759	0.667				0.966	0.25–3.737	0.96			
Female	Reference																	
Age (years)																		
≥ 61	1.124	0.554–2.28	0.747				0.922	0.466–1.825	0.816				0.896	0.259–3.094	0.862			
< 61	Reference																	

*Abbreviations*: *anti-HBc* antibodies to hepatitis B core antigen, *anti-HBs* antibodies to hepatitis B surface antigen, *HBsAg* hepatitis B surface antigen, *NLR* neutrophil to lymphocyte ratio, *CA19-9* cancer antigen 19-9, *CEA* carcinoembryonic antigen, *ALT* alanine transaminase, *AST* aspartate transaminase, *ALP* alkaline phosphatase, *PLT* platelets

*Significant at *P* < 0.05

## Discussion

In this study, we used anti-HBc, which is different from HBsAg used in previous studies, as a marker of HBV infection [17, 18], because using HBsAg as the criteria of grouping may lead to the grouping mistake by deeming occult hepatitis B infection as non-infected and generate significant deviation. After people get infected by HBV, a covalently closed circular DNA form (cccDNA) is deposited to serve as a template for the transcription of all HBV RNAs, the production of progeny virus [19] and conduct consistent inflammation of human body [20]. The cccDNA can be detected frequently in the liver of the HBsAg negative phase patients [21]. HBV can be hardly cured by available antivirals, because neither cccDNA nor relax circular DNA (rcDNA) is affected during the anti-viral process [19]. For this reason, the European Association for the Study of the Liver (EASL) and American Association for the Study of Liver Diseases (AASLD) termed the therapeutic goal of chronic hepatitis B (CHB) “functional cure,” while the true cure is the elimination of cccDNA [21, 22]. Several studies have proved even HBV related hepatocellular carcinoma and complications of cirrhosis [23–25] can still occur in the resolved HBV patients when they are receiving immunosuppressive therapy.

Anti-HBc, generated by humoral immunity, is highly stable. After acute HBV infection, IgM class antibodies are firstly observed in the host’s organism, and then anti-HBc IgG begins to appear. Over time, IgM levels gradually decline until they cannot be detected, and IgG can persist for 10 or even more than 20 years [11]. Anti-HBc also has a relatively high specificity; it is produced in the presence of HBV infection rather than a serological response to HBV vaccination, while anti-HBs can appear in both situations. Therefore, anti-HBc was chosen to use as a serum marker for HBV infection in our study.

Our data showed no statistical difference between the anti-HBc positive group and anti-HBc negative group about the incidence of CRLM. Prior to this, Utsunomiya T et al. analyzed the association of hepatitis virus infection and the incidence of CRLM in 1999. He found that the incidence of CRLM of the infected group was significantly lower than non-infected group (8.1% vs. 21.2%, *P* < .05) [26]. However, the study did not analyze HBV and HCV separately. In 2001, Song E et al. found that the incidence of CRLM in patients with HBV infection was significantly lower than that in patients who are not infected (13.5% vs. 27.1, *P* < .05), and the prognosis of infected patients was better [27].

Some studies reached the opposite conclusion. Huo T et al. performed a cross-sectional study about CRC patients and reported that chronic HBV infection increased the risk of CRLM. Also, the study found that the incidence of CRLM of the HBeAg-positive patients was higher than it of the HBeAg-negative patients [18]. It indicated that activated replication of HBV could increase the risk of CRLM, although there are not any significant differences identified. A similar phenomenon was found in the study of Wei X et al. where the pancreatic cancer was included as the object of the study instead of CRC, and the phenomenon that the rate of liver metastases in CHB patients was higher than both uninfected patients and patients with resolved HBV infection (61.1% vs. 33.9%, *P* < 0.05, and 61.1% vs. 28.7%, *P* < 0.05, respectively) [28] was observed.

Our outcome differs from previous studies. The probably reason is the recognition of HBV infection in our study differs from other studies in which “HBV infection” was characterized by positive HBsAg. Therefore, the composition of “HBV-infected patients” in this study varies from other studies. Also, HBV replication activity of anti-HBc positive cases varies from the “HBV-infected” cases in previous studies. Additionally, both Wei X and Huo T have observed a likely correlation between the activity of HBV replication and liver metastases of malignant tumors.

In 1974, Hoofnagle JH et al. [13] proposed that anti-HBc, particularly in high titers, would reflect active replication of HBV. In 1992, Hisao Iizuka et al. [29] observed that the detection rate of HBV DNA in blood units with high-titer anti-HBc was higher than that with low-titer anti-HBc, which conforms with Hoofnagle’s conclusion. In general, the cccDNA which serves as the template for HBV replication and transcription can directly reflect the intrahepatic activity of HBV replication. Unfortunately, the liver biopsy, which is indispensable for the quantitative analysis of cccDNA, cannot be carried out in this study.

It is worth mentioning that Caviglia GP et al. [12] reported the correlation between anti-HBc and cccDNA. They found that high-titer anti-HBc was associated with the finding of intrahepatic HBV cccDNA, while low-titer anti-HBc could exclude the presence of cccDNA. So, we conducted a subset analysis concerning the titer of anti-HBc, in which anti-HBc positive group was divided into the high-titer anti-HBc group and low-titer anti-HBc group. We found that higher titer anti-HBc predicts a higher risk of CRLM and worse survival. The possible explanations for this phenomenon are listed below. (1) HBV may promote the development of CRC by HBx-mediated miR-34a downregulation which was observed in the research about the HBV-related HCC [30], and the miR-34 family was found to have antitumor activity, especially miR-34a, which was reported to promote CRC when it was downregulated [31, 32]. (2) HBV may promote CRC by altering the human gut microbiome which plays an important role in the development of CRC [33–35]. Due to the existence of the hepato-intestinal axis, studies have shown that HBV affects the development of HCC by changing the intestinal flora [36], but whether this mechanism can be applied to CRLM needs to be further studied. However, Qin N et al. [37]. reported that in the patients with liver cirrhosis, the abundance of Lachnospiraceae which could inhibit the development of CRC by producing butyric acid was decreased [35, 38], while there was an increase in the *Fusobacterium* which could promote CRC by upregulating tumor-associated macrophages (TAMs) [39, 40].

Some previous studies suggest that cirrhosis could inhibit CRLM. As early as 1975, Hamaya K et al. [41] conducted several autopsies and observed the incidence of liver metastases in cirrhotics was lower than non-cirrhotics (26.3% vs. 43.2%). A series of subsequent studies have reached similar conclusions [42, 43]. This is probably because under the stimulation of pathogenic factors such as inflammation, the liver gradually gets fibrotic until pseudo-lobules are formed, which leads to the tortuous deformation of intrahepatic small vessels, and afterward hemodynamics changes of the liver occur. Moreover, the sinusoid capillarization causes transformations of adhesion factors and extracellular matrix, which is not conducive to the growth of tumor cells in the liver. Furthermore, the expression level of MMP inhibitors in the cirrhotic liver is also higher, which may be another component that inhibits the formation of CRLM in cirrhotic cases. However, there is no more convincing explanation for this phenomenon so far [43].

In this study, we used FIB-4 as predictors of fibrosis to evaluate its impact on CRLM. But no statistical difference was identified. This is probably because in spite of 81 cases with FIB-4 > 1.45 was identified, 77 cases of them were in the 1.45–3.25 interval which was not clearly defined so far [44], and only 4 cases were in the FIB-4 > 3.25 interval which corresponds to F3 and F4 (also known as advanced fibrosis) in the Metavir stage classification system. Besides, as an indirect indicator of liver fibrosis [14, 15], the accuracy of FIB-4 is not as good as the liver biopsy. It is worth mentioning that the liver biopsy, an invasive inspection, was not routinely performed before the colorectomy, except that cirrhosis-related symptoms occurred.

We found that NLR ≥ 3.4, CA19-9 > 37 U/ml, and stage III were the predictors of adverse outcomes, which strongly support previous reports [45–54] and clinical experience.

There are several limitations in our approach. First, this is a retrospective single-institution study with relatively small sample size and multi-center, prospective, large-scale trials are needed in the future. Second, some clinical data such as serum HBV DNA, intrahepatic HBV DNA, and cccDNA were not available in the study. As a retrospective study, we are unable to obtain these data. Finally, we did not analyze the usage of antivirals which may affect the outcomes.

Of note, we developed a basis for understanding the relationship between HBV infection, prognosis, and liver metastases in CRC patients using anti-HBc as the criterion for distinguishing HBV infection, avoiding errors generated by using HBsAg as the criterion for grouping. Moreover, we first established a link between the anti-HBc titer and the prognosis and liver metastases of CRC patients. Notably, we excluded patients who were infected with HAV, HCV, and HEV and patients with liver metastases before surgery to ensure the uniformity of baseline data to the greatest extent.

## Conclusion

Our data demonstrated that higher titer anti-HBc predicts a higher risk of liver metastases and worse survival in anti-HBc positive patients with colorectal cancer undergoing curative surgical resection, which implies the close relationship between highly active replication of HBV and occurrence of CRLM. This gives us some enlightenment on the management of CRC patients. For the management of CRC, we should pay more attention to the status of HBV, especially those whose serum anti-HBc are above 8.8 S/CO, because this may pose great impact to improving the prognosis of such patients. Moreover, we can draw up more personalized follow-up plans for patients based on their anti-HBc titers.

## Supplementary Information

**Additional file 1: Supplementary Figure 1.** Flow diagram of the retrospective analysis with adequate data.

**Additional file 2: Supplementary Figure 2.** X-tile plots of the anti-HBc. Notes: X-tile plots showing χ2 values with cut-off points to generate the low-titer and high-titer anti-HBc subgroups. (A) The optimal cutoff value of the anti-HBc was 8.8 at the maximum χ2 value of 18.44. (B) Histogram of the entire cohort divided into low-titer anti-HBc and high-titer anti-HBc subgroups according to the optimal cutoff value of 8.8. Blue bars represent the low-titer anti-HBc group, and gray bars represent the high-titer anti-HBc group.

## Data Availability

The datasets used and/or analyzed during the current study are available from the corresponding author on reasonable request.
