# Supplementary figures and images for: Higher titer hepatitis B core antibody predicts a higher risk of liver metastases and worse survival in patients with colorectal cancer
Source: World J Surg Oncol. 2021 Aug 26;19:251. doi: 10.1186/s12957-021-02369-1 (PMC8394189; doi:10.1186/s12957-021-02369-1)

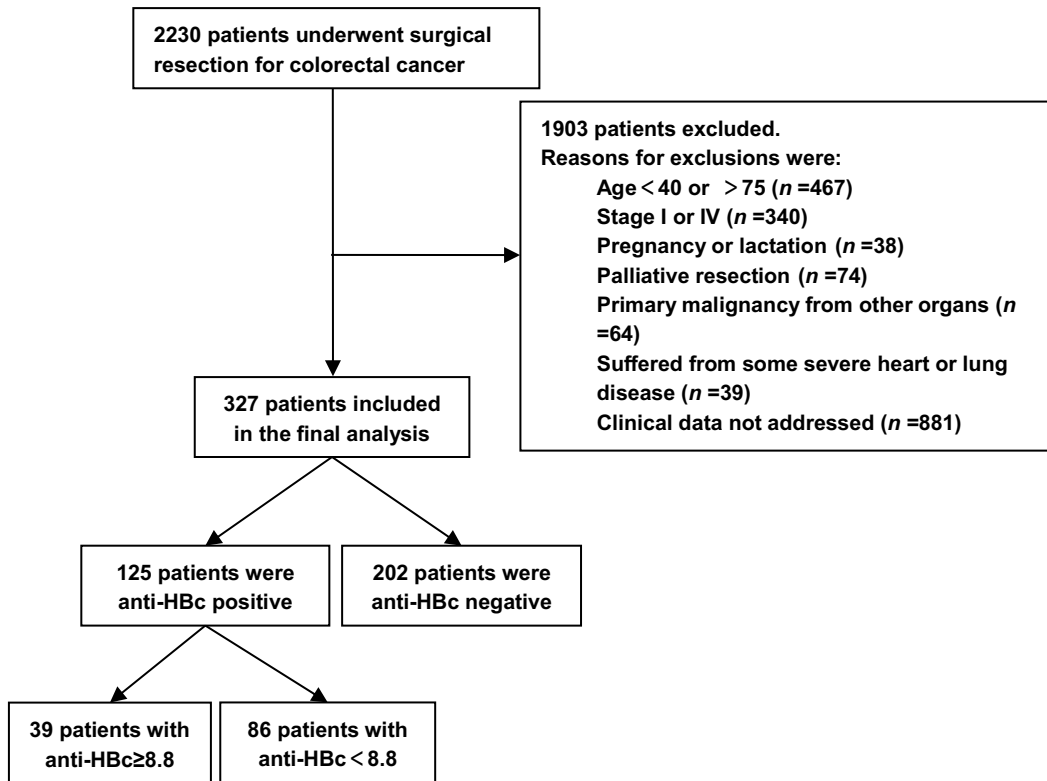

Supplement: Supplementary file 1 — Additional file 1: Supplementary Figure 1. Flow diagram of the retrospective analysis with adequate data. [file 12957_2021_2369_MOESM1_ESM.pdf]
