# Supplementary material for: Higher titer hepatitis B core antibody predicts a higher risk of liver metastases and worse survival in patients with colorectal cancer
Source: World J Surg Oncol. 2021 Aug 26;19:251. doi: 10.1186/s12957-021-02369-1 (PMC8394189; doi:10.1186/s12957-021-02369-1)

A

Pt No	% Total	Events	Rate	Rank	Range
87	69.6	17	19.54	0 to 82	1.02 thru 8.80
38	30.4	21	55.26	83 to 118	8.81 thru 14.11
125	100	38	30.4		1.02 thru 14.11
Miller-Seigmund P		< .001		Max: < .001	
Chi-sq Hi/Lo		18.44		Max: 18.44	
Relative Risk 1 vs 2		1.00/2.83			

B

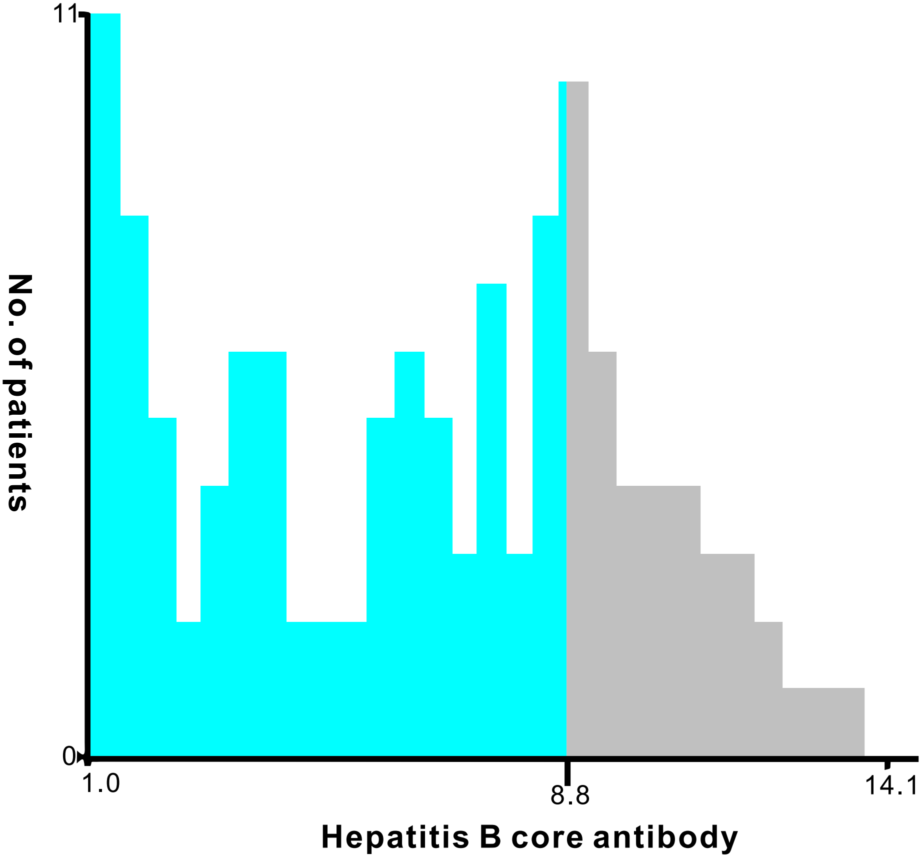

Supplement: Supplementary file 2 — Additional file 2: Supplementary Figure 2. X-tile plots of the anti-HBc. Notes: X-tile plots showing χ2 values with cut-off points to generate the low-titer and high-titer anti-HBc subgroups. (A) The optimal cutoff value of the anti-HBc was 8.8 at the maximum χ2 value of 18.44. (B) Histogram of the entire cohort divided into low-titer anti-HBc and high-titer anti-HBc subgroups according to the optimal cutoff value of 8.8. Blue bars represent the low-titer anti-HBc group, and gray bars represent the high-titer anti-HBc group. [file 12957_2021_2369_MOESM2_ESM.pdf]
